# Cryptotaenia japonica extract attenuates glucocorticoid-induced muscle atrophy via regulation of the AKT/mTOR–FOXO/E3 ubiquitin ligase axis

**DOI:** 10.3389/fphys.2026.1860064

**Published:** 2026-07-16

**Authors:** Da-In Choi, HuiJun Lee, Seokhoon Heo, Subin Cho, Pansoo Kim, Chul-Yung Choi

**Affiliations:** 1 Well-Aging Medicare & City G-LAMP Project Group, Chosun University, Gwangju, Republic of Korea; 2 Department of Integrative Biological Sciences & BK21 FOUR Educational Research Group for Age-Associated Disorder Control Technology, Chosun University, Gwangju, Republic of Korea; 3 Orgasis Corp., Suwon, Gyeonggi, Republic of Korea; 4 Department of Biomedical Science, Chosun University, Gwangju, Republic of Korea

**Keywords:** Cryptotaenia japonica, FoxO signaling, glucocorticoids, mTOR pathway, muscle atrophy, sarcopenia

## Abstract

Sarcopenia, characterized by progressive loss of skeletal muscle mass and function, is exacerbated by chronic glucocorticoid exposure, which activates catabolic signaling pathways and accelerates muscle protein degradation. Although *Cryptotaenia japonica* Hassk (Apiaceae) has been reported to possess antioxidant and anti-inflammatory properties, its role in glucocorticoid-induced muscle atrophy remains unclear. In this study, we investigated the myoprotective effects of *Cryptotaenia japonica* extract (CJE) using both *in vitro* and *in vivo* models. C2C12 myotubes were treated with dexamethasone (Dex, 10 μM) in the presence or absence of CJE (10–100 μg/mL), and key signaling pathways were analyzed by Western blotting and confocal microscopy. *In vivo*, Dex-induced muscle atrophy was established in ICR mice, followed by oral administration of CJE (200 mg/kg/day). Muscle tissues were evaluated for protein expression, histological alterations, and serum GDF-8 levels. CJE treatment attenuated Dex-induced upregulation of the E3 ubiquitin ligases MuRF-1 and FBX32 and inhibited FOXO1 nuclear translocation in C2C12 myotubes. In dexamethasone-treated mice, CJE restored mTOR phosphorylation and normalized the dexamethasone-induced dysregulation of AKT phosphorylation, while suppressing the FOXO/E3 ubiquitin ligase catabolic axis. In Dex-treated mice, CJE reduced the expression of FOXO3a, MuRF-1, and FBX32, preserved muscle fiber architecture, and increased muscle fiber cross-sectional area. Furthermore, CJE modulated circulating GDF-8 levels associated with muscle atrophy. Collectively, these findings demonstrate that CJE mitigates glucocorticoid-induced muscle atrophy by coordinately regulating the AKT/mTOR–FOXO/E3 ubiquitin ligase signaling axis. These results suggest that CJE may serve as a promising natural therapeutic candidate for the prevention of sarcopenia and muscle-wasting conditions.

## 1 Introduction

Sarcopenia, defined as the age-related progressive loss of skeletal muscle mass, strength, and function, represents a major public health challenge with profound implications for mobility, metabolic health, and quality of life in aging populations (Wu et al., 2023). The molecular networks underlying sarcopenia involve complex interactions among protein synthesis, degradation, mitochondrial dysfunction, satellite cell dysfunction, and inflammatory signaling (Altun et al., 2010; Wang et al., 2020). Its prevalence increases markedly with age, affecting approximately 10–27% of individuals over 60 years and up to 50% of those over 80 years (Sakuma and Yamaguchi, 2012). Beyond natural aging, sarcopenia can be accelerated by pathological conditions including chronic inflammation, metabolic disorders, and prolonged glucocorticoid exposure (Bowen et al., 2015).

Glucocorticoids, widely prescribed for their anti-inflammatory and immunosuppressive properties, exert significant adverse effects on skeletal muscle when used chronically (Egerman and Glass, 2014). Glucocorticoid-induced myopathy is characterized by preferential atrophy of type II fibers, progressive weakness, and impaired functional capacity (Pereira and Freire de Carvalho, 2011), and arises from interconnected pathways that disrupt the balance between muscle protein synthesis and degradation (Morgan et al., 2009; Schakman et al., 2012; Schakman et al., 2013). Dexamethasone (Dex), a synthetic glucocorticoid, has been widely used to model muscle atrophy in both cultured myotubes and animals, providing a well-established platform for evaluating anti-atrophic interventions (Menconi et al., 2008).

A central mechanism in glucocorticoid-induced atrophy is activation of the Forkhead box O (FOXO) transcription factors, particularly FOXO1 and FOXO3a (Zhao et al., 2007). Under anabolic conditions, FOXO proteins are phosphorylated by Akt and retained in the cytoplasm in an inactive, 14-3-3–bound state (Brunet et al., 1999; Milan et al., 2015). Glucocorticoid exposure disrupts this regulation, promoting FOXO dephosphorylation, nuclear translocation, and transcription of atrophy-related genes (Chen et al., 2022). Nuclear FOXO upregulates the muscle-specific E3 ubiquitin ligases Atrogin-1 (MAFbx/FBX32) and muscle RING-finger protein 1 (MuRF-1) (Bodine et al., 2001; Gomes et al., 2001; Sato et al., 2017), which target myofibrillar proteins for ubiquitin–proteasome degradation (Gumucio and Mendias, 2013; Sandri, 2013). Suppression of these atrogenes prevents Dex-induced myotube atrophy, confirming their causal role (Castillero et al., 2013). Concurrently, glucocorticoids suppress anabolic signaling through the IGF-1/PI3K/Akt/mTOR axis, reducing protein synthesis while enhancing degradation (Schiaffino and Mammucari, 2011; Laplante and Sabatini, 2012; Saxton and Sabatini, 2017); the resulting imbalance drives rapid muscle wasting (Rommel et al., 2001).

Given the clinical burden of glucocorticoid-induced sarcopenia and the limited pharmacological options, plant-derived compounds capable of modulating multiple muscle-homeostatic pathways have attracted growing interest. Flavonoids and other polyphenols counteract muscle atrophy through modulation of the Akt/mTOR/FOXO axis, attenuation of oxidative stress, and suppression of inflammatory signaling (Kim and Hwang, 2020; Nikawa et al., 2021), and dietary nutraceuticals have emerged as a promising strategy against glucocorticoid-induced atrophy (Wang et al., 2021; Lee et al., 2022). Several botanical extracts have demonstrated protective effects against experimental skeletal muscle injury and muscle wasting in preclinical models, including chemotherapy-associated muscle dysfunction, supporting the rationale for evaluating additional phytochemical-rich extracts (Wu et al., 2022; Wu et al., 2023b; Wu et al., 2023a).

*Cryptotaenia japonica* Hassk. (Apiaceae), commonly known as Japanese honewort or mitsuba, is a perennial herb widely distributed in East Asia and traditionally used in folk medicine. Its aerial parts contain diverse bioactive constituents including phenolic acids, flavonoids, coumarins, and polyacetylenes that underlie its reported antioxidant and anti-inflammatory activities (Lu et al., 2018). Despite these properties, the muscle-protective potential of C. japonica in glucocorticoid-induced sarcopenia has not been investigated. The present study therefore evaluated the myoprotective effects of C. japonica extract (CJE), in which chlorogenic acid was detected as a marker compound, using complementary *in vitro* and *in vivo* models.

## 2 Materials and methods

### 2.1 Preparation of *Cryptotaenia japonica* extract

**Figure 1:**
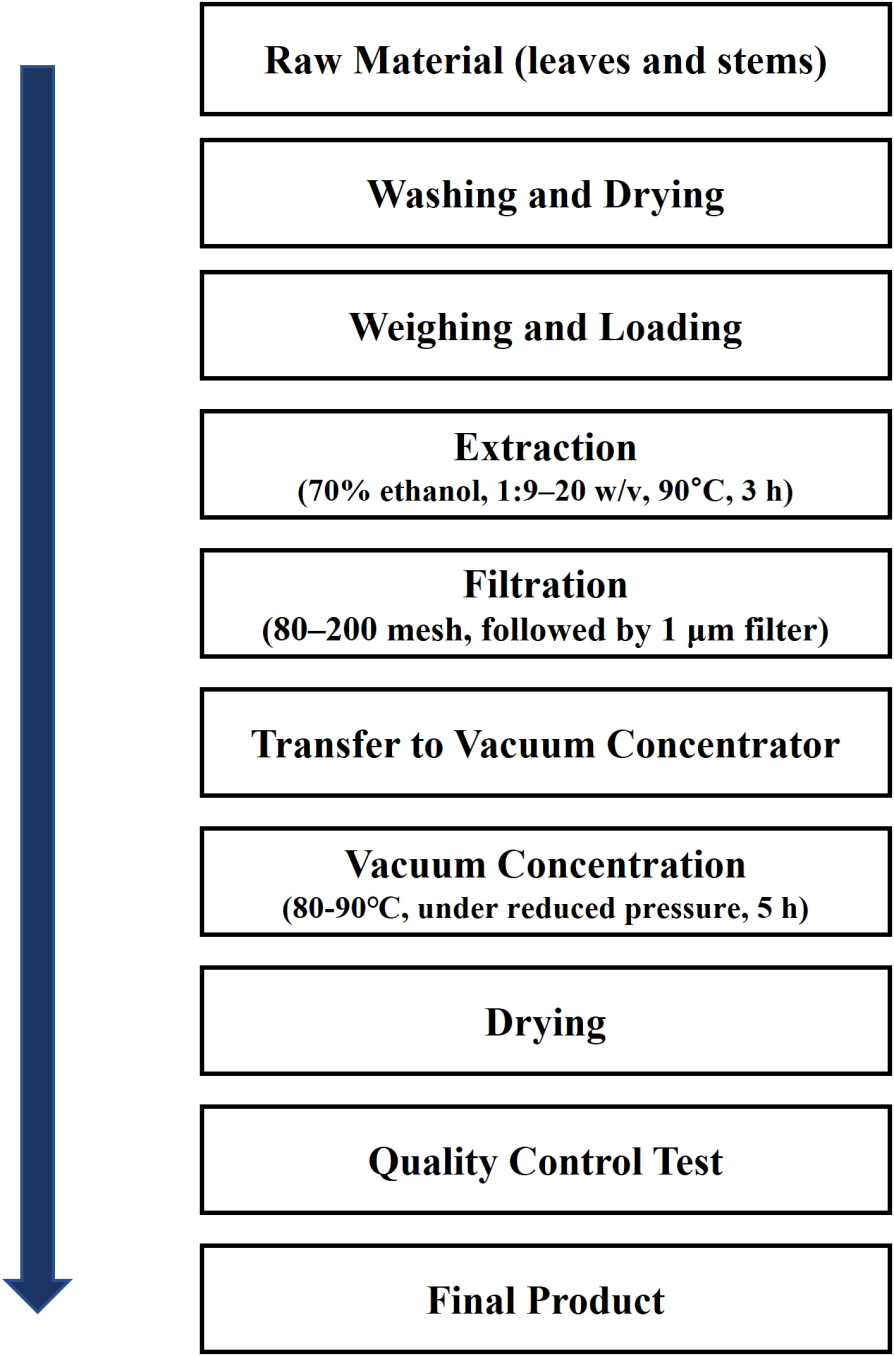
Manufacturing process of *Cryptotaenia japonica* extract provided by Orgasis Corp. The extract was prepared from leaves and stems using 70% ethanol extraction, followed by filtration, vacuum concentration, drying, and quality control.

*Cryptotaenia japonica* extract (CJE) was provided by Orgasis Corp. (Suwon, Republic of Korea). The extract was prepared from the leaves and stems of *Cryptotaenia japonica* Hassk. (Apiaceae). The manufacturing process of the extract is illustrated in **Figure 1**. The CJE powder was stored at -20 °C until use and dissolved in dimethyl sulfoxide (DMSO) for *in vitro* experiments or sterile water for *in vivo* administration. The final DMSO concentration in cell culture experiments did not exceed 0.1% (v/v).

### 2.2 UHPLC–MS analysis of chlorogenic acid

Chlorogenic acid in *Cryptotaenia japonica* extract (CJE) was analyzed by an external certified analytical facility using UHPLC–MS/MS in multiple reaction monitoring (MRM) mode. Chromatographic separation was performed on a Nexera X2 UHPLC system coupled to an LCMS-8050 mass spectrometer (Shimadzu, Kyoto, Japan). Separation was achieved using an ACQUITY BEH C18 column (100 × 2.1 mm, 1.7 μm; Waters, Milford, MA, USA). The mobile phases consisted of (A) water containing 0.1% formic acid and (B) methanol. The gradient program was as follows: 0–4.0 min, 20–80% B; 4.0–7.0 min, 80% B; 7.1–10.0 min, re-equilibration at 20% B. The flow rate was 0.3 mL/min and the injection volume was 2 μL.

Mass spectrometric detection was performed in positive electrospray ionization (ESI+) mode with a spray voltage of 4500 V, nebulizer gas pressure of 20 psi, and source temperature of 200 °C. Chlorogenic acid was monitored using the precursor ion at *m/z* 353.00 and product ions at *m/z* 191.35, 85.05, and 92.95. Identification was based on retention time and characteristic fragmentation patterns, and quantification was performed using an external calibration curve generated from authentic chlorogenic acid standards (10–1000 ng/mL). The calibration curve showed excellent linearity (R² = 0.9993).

### 2.3 Cell culture and myogenic differentiation

The Murine C2C12 myoblasts (American Type Culture Collection, Manassas, VA, USA) were cultured in Dulbecco’s Modified Eagle Medium (DMEM; Gibco, Grand Island, NY, USA) supplemented with 10% fetal bovine serum (FBS; Gibco) and 1% penicillin-streptomycin at 37 °C in a humidified atmosphere containing 5% CO2. To induce myogenic differentiation, C2C12 myoblasts at 80-90% confluence were switched to differentiation medium consisting of DMEM supplemented with 2% horse serum (Gibco) and 1% penicillin-streptomycin. Differentiation medium was changed every 48 hours, and cells were maintained under differentiation conditions for 5–7 days to allow formation of multinucleated myotubes.

### 2.4 Cell viability assay

Cell viability was assessed using the MTS (3-(4,5-dimethylthiazol-2-yl)-5- (3-carboxymethoxyphenyl)-2-(4- sulfophenyl)-2H-tetrazolium) assay (MTS; Promega, Madison, WI, USA). C2C12 myoblasts were treated with various concentrations of CJE for 24 hours, and cell viability was measured according to the manufacturer’s instructions. Results were expressed as a percentage of control.

### 2.5 *In Vitro* dexamethasone-induced atrophy model and CJE treatment

Fully differentiated C2C12 myotubes were treated with dexamethasone (Dex; Sigma-Aldrich, St. Louis, MO, USA) at 10 μM to induce muscle atrophy, as established in our previous study (Schiaffino and Mammucari, 2011). CJE was added to the culture medium at concentrations of 10, 25, 50, or 100 μg/mL simultaneously with Dex treatment. Control myotubes received vehicle (0.1% DMSO) only. After 24–48 hours of treatment, myotubes were harvested for morphological analysis, protein extraction, or immunofluorescence staining (Rommel et al., 2001).

### 2.6 Western blot analysis

Cells Protein lysates were prepared from gastrocnemius muscle tissues and C2C12 cells using RIPA buffer containing protease and phosphatase inhibitors (Thermo Fisher Scien-tific, Waltham, MA, USA). Protein concentrations were determined using the Bradford (Bio-Rad, Hercules, CA, USA). Equal amounts of protein (30–50 µg) were separated by SDS–PAGE and transferred onto PVDF membranes (Millipore, Billerica, MA, USA). Membranes were blocked with 5% non-fat dry milk and incubated overnight at 4 °C with the following primary antibodies: phospho-mTOR(#5536; Cell Signaling Technology, Danvers, MA, USA), total mTOR(#2983; Cell Signaling Technology), phospho-Akt (Ser473, #4060; Cell Signaling Technology), total Akt (#9272; Cell Signaling Technology), FoxO1 (#2880; Cell Signaling Technology), Phospho-FoxO1 (Thr24)/FoxO3a (THR32, #9464; Cell Signaling Technology), total FOXO3α (#12829; Cell Signaling Technology), FBX32 (#ab168372; Abcam, Cambridge, UK), MuRF-1 (C-2; sc-398608, Santa Cruz Biotechnology, Dallas, TX, USA), and GAPDH (#2118; Cell Signaling Technology). After washing, membranes were incubated with horseradish peroxidase (HRP)-conjugated secondary antibodies (Cell Signaling Technology) for 1 h at room temperature. Protein bands were visualized using enhanced chemiluminescence (Super Signal™ West Pico PLUS, #34580; Thermo Fisher Scientific, Waltham, MA, USA) and quantified by densitometry using ImageJ software (NIH, Bethesda, MD, USA).

### 2.7 Immunofluorescence microscopy

For FOXO1 subcellular localization analysis, differentiated C2C12 myotubes grown on glass coverslips were fixed with 4% paraformaldehyde, permeabilized with 0.1% Triton X-100, and blocked with 3% BSA in PBS. Cells were incubated overnight at 4 °C with anti-FOXO1 primary antibody (1:200; Cell Signaling Technology), followed by Alexa Fluor 594-conjugated secondary antibody (1:500; Invitrogen). Nuclei were counterstained with DAPI. Images were captured using an inverted fluorescence microscope (Nexcope NIB610FL, Ningbo Yongxin Optics Co., Ltd., China) at 40× magnification.

### 2.8 Animal experiments

All animal experiments were conducted in accordance with the guidelines of the Institutional Animal Care and Use Committee (IACUC) of Chosun University (approval number: CIACUC2025-A0023) and conducted in accordance with its ethical guideline. Male ICR mice (8–10 weeks old, 25–30 g) were obtained from a certified supplier and housed under standard laboratory conditions (12 h light/dark cycle, 22 ± 2 °C, 50–60% humidity).

#### 2.8.1 Dexamethasone-induced muscle atrophy model in mice

ICR mice were randomly divided into three groups (n = 4~6 per group): (1) Control (vehicle, sterile water, oral gavage); (2) Dex (dexamethasone 10 mg/kg/day, i.p.); and (3) Dex + CJE (dexamethasone 10 mg/kg/day, i.p., plus CJE 200 mg/kg/day, oral gavage). The dose of CJE (200 mg/kg/day) was selected based on preliminary dose-finding experiments demonstrating acceptable tolerability and biological activity. Dexamethasone was administered daily for 14 consecutive days. CJE was administered 1 hour before dexamethasone injection. Body weight was monitored daily.

### 2.9 Tissue collection and histological analysis

At the end of the experimental period, animals were fasted for 12 hours, after which blood was collected for serum preparation. Gastrocnemius (calf) muscles were dissected, weighed, and either snap-frozen in liquid nitrogen or fixed in 10% neutral buffered formalin. Formalin-fixed tissues were paraffin-embedded, sectioned at 5 μm, and stained with hematoxylin and eosin (H&E) following standard protocols. Muscle fiber cross-sectional area (CSA) was measured from at least 300 individual fibers per animal using ImageJ software. (National Institutes of Health, Bethesda, MD, USA).

### 2.10 Serum GDF-8 (myostatin) measurement

Serum concentrations of growth differentiation factor-8 (GDF-8/myostatin) were measured using a commercially available ELISA kit (#DGDF80, R&D Systems, Minneapolis, MN, USA) according to the manufacturer’s instructions (Kim and Hwang, 2020). All samples were analyzed in triplicate, and results were adjusted for dilution factors.

### 2.11 Statistical analysis

All experiments were performed in at least three independent replicates. Data are presented as mean ± SEM. Statistical comparisons were performed using one-way ANOVA followed by Tukey’s *post hoc* test for multiple comparisons, or Student’s t-test for two-group comparisons, using JASP software (version 0.19.3; University of Amsterdam, Amsterdam, The Nether-lands). Statistical significance: *P < 0.05, **P < 0.01, ***P < 0.001 vs. control; # *P* < 0.05, ## *P* < 0.01, ### *P* < 0.001 vs. dexamethasone group.

## 3 Result

### 3.1 Preparation of *Cryptotaenia japonica* extract

Cryptotaenia japonica extract (CJE) used in this study was provided by Orgasis Corp. The manufacturing process is summarized in **Figure 1**. The extract was prepared from the leaves and stems of Cryptotaenia japonica using 70% ethanol extraction, followed by filtration, vacuum concentration, drying, and quality control to obtain the final product. This process ensured the preparation of a standardized extract for subsequent *in vitro* and *in vivo* experiments.

### 3.2 Phytochemical characterization of CJE and identification of chlorogenic acid

**Figure 2:**
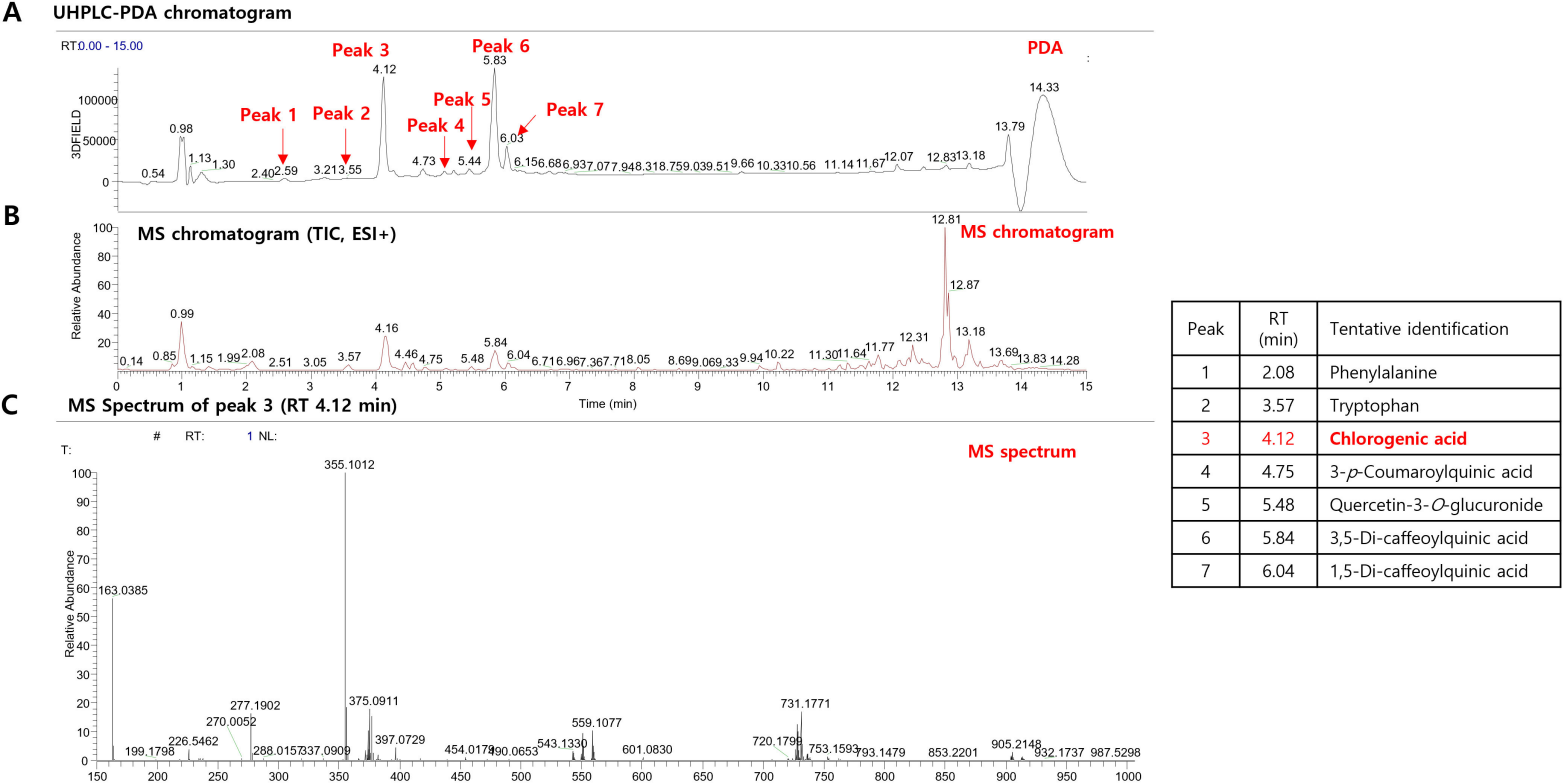
Representative UHPLC–PDA fingerprint and LC–MS characterization of Cryptotaenia japonica extract. **(A)** UHPLC–PDA chromatogram showing the overall phytochemical fingerprint of the extract. Multiple chromatographic peaks were detected, with Peak 3 (RT = 4.12 min) identified as chlorogenic acid, a representative marker compound. **(B)** Total ion chromatogram (TIC) acquired in positive ESI mode. **(C)** Mass spectrum of Peak 3 confirming chlorogenic acid. Tentative assignments of the major peaks identified by LC–MS are summarized in the accompanying table.

UHPLC–MS analysis was performed to characterize the phytochemical profile of CJE. As shown in **Figure 2A**, the UHPLC–PDA chromatogram revealed multiple chromatographic peaks, indicating that CJE contains diverse phytochemical constituents. Among these peaks, Peak 3 (RT = 4.12 min) was identified as chlorogenic acid and selected as a representative marker compound. The identity of chlorogenic acid was further confirmed by the total ion chromatogram (**Figure 2B**) and the corresponding mass spectrum (**Figure 2C**). Tentative assignments of the major chromatographic peaks identified by LC–MS are summarized in **Figure 2**.

External LC–MS/MS (MRM) analysis further identified chlorogenic acid with a precursor ion at *m/z* 353.00 and major product ions at *m/z* 191.35, 85.05, and 92.95 at a retention time of 2.740 min. The calibration curve demonstrated excellent linearity over the concentration range of 10–1000 ng/mL (R² = 0.9993), supporting reliable detection and quantification of chlorogenic acid in CJE.

### 3.3 Effects of CJE on cell viability in C2C12 myoblasts

**Figure 3:**
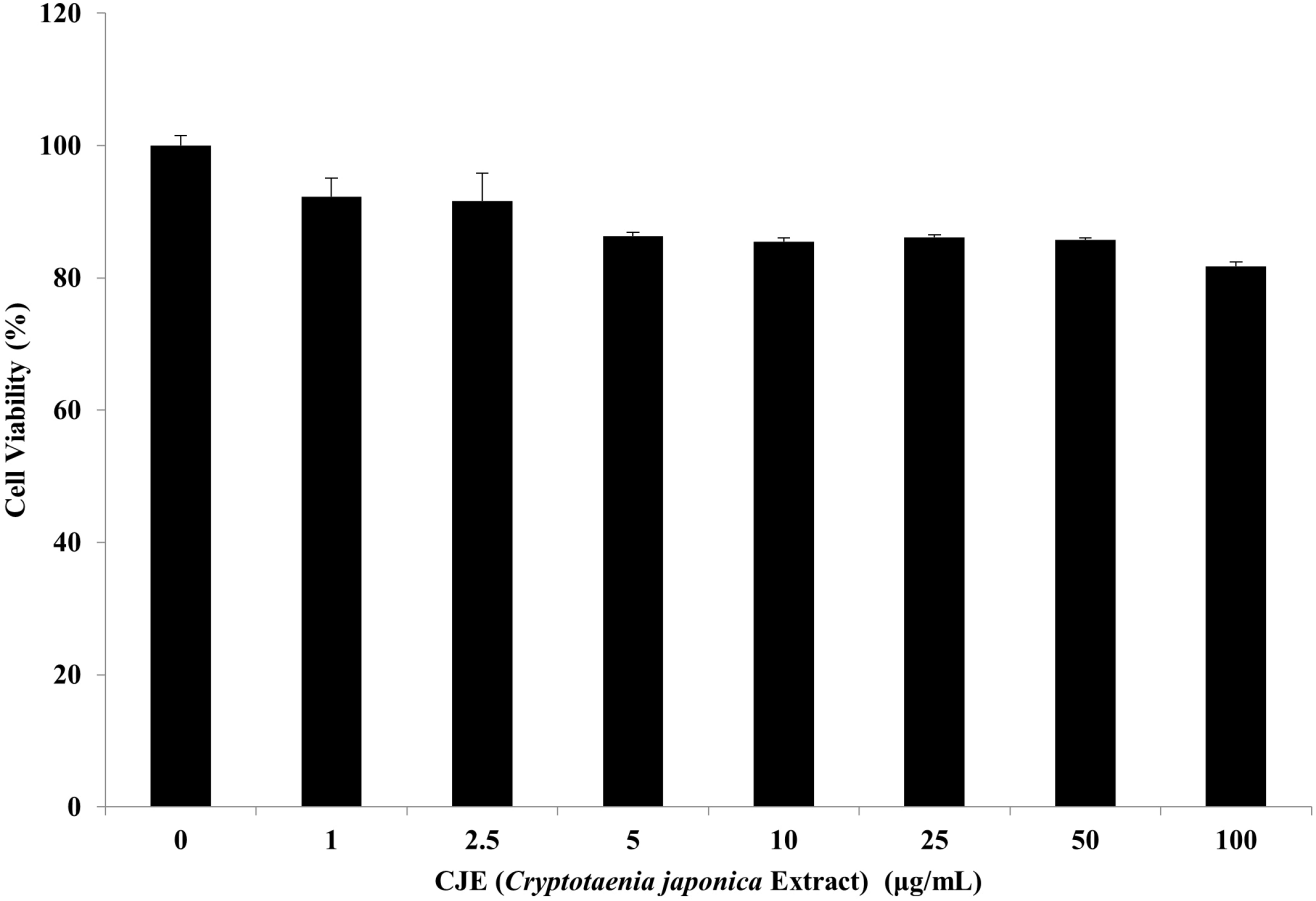
Effect of Cryptotaenia japonica extract (CJE) on C2C12 myoblast viability. C2C12 myoblasts were treated with CJE (1–100 μg/mL) for 24 h. Cell viability was determined by MTS assay and expressed as a percentage of the untreated control. Data are presented as mean ± SEM (n = 3).

To evaluate the cytocompatibility of Cryptotaenia japonica extract (CJE), undifferentiated C2C12 myoblasts were treated with CJE at concentrations ranging from 1 to 100 μg/mL for 24 h. As shown in **Figure 3**, CJE caused only a modest reduction in cell viability, which plateaued at approximately 81–86% at concentrations ≥5 μg/mL. The mean viability values were 92.4%, 91.6%, 86.3%, 85.4%, 86.1%, 85.7%, and 81.5% at 1, 2.5, 5, 10, 25, 50, and 100 μg/mL, respectively. Because cell viability remained above 80% at all tested concentrations, CJE was considered non-cytotoxic under the present experimental conditions. These concentrations were therefore used for subsequent mechanistic studies in differentiated C2C12 myotubes.

### 3.4 Effects of CJE on basal anabolic and protein turnover-related signaling proteins in C2C12 myotubes

**Figure 4:**
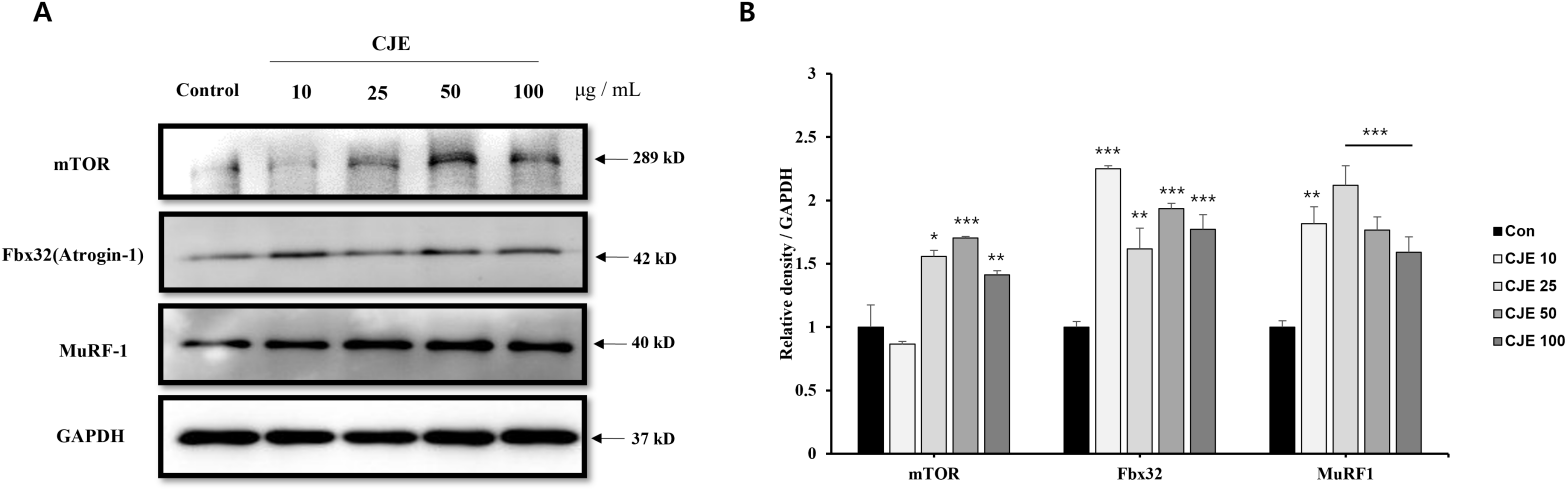
Effect of CJE on basal expression of mTOR and muscle-specific E3 ubiquitin ligases in C2C12 myotubes. C2C12 myotubes were treated with CJE at concentrations of 10, 25, 50, and 100 μg/mL for 24 h. Protein expression levels of mTOR, FBX32 (Atrogin-1) and MuRF-1 were determined by Western blotting. GAPDH was used as a housekeeping protein. **(A)** Representative Western blot images showing the indicated proteins in each group. GAPDH was used as a loading control. **(B)** Densitometric analysis of protein expression normalized to GAPDH. Data are presented as mean ± SEM (n = 3). Statistical comparisons were performed using one-way ANOVA followed by Dunnett’s *post hoc* test. *p < 0.05, **p < 0.01, ***p < 0.001 vs. Control.

To examine the effect of CJE on basal muscle signaling under non-atrophic conditions, differentiated C2C12 myotubes were treated with CJE (10–100 μg/mL) for 24 h. Representative Western blot images and densitometric analyses are shown in **Figures 4A, B**, respectively. CJE increased total mTOR expression in a concentration-dependent manner, indicating enhanced anabolic signaling. Notably, the muscle-specific E3 ubiquitin ligases FBX32 (Atrogin-1) and MuRF-1 were also elevated relative to the untreated control. The concurrent upregulation of both the anabolic regulator (mTOR) and protein-turnover markers (E3 ubiquitin ligases) suggests that, under basal conditions, CJE may promote overall protein turnover and remodeling rather than exert a purely anti-catabolic effect. The functionally relevant anti-atrophic activity of CJE was therefore evaluated under dexamethasone-induced catabolic conditions.

### 3.5 CJE attenuates dexamethasone-induced upregulation of E3 ubiquitin ligases in C2C12 myotubes

**Figure 5:**
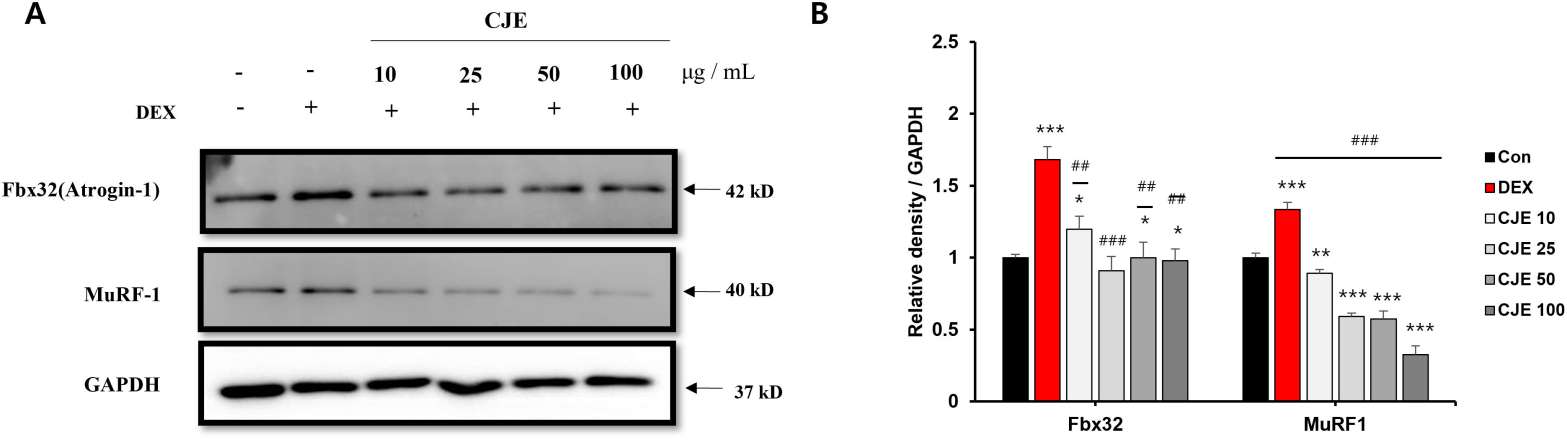
CJE attenuates dexamethasone-induced upregulation of E3 ubiquitin ligases in C2C12 myotubes. Differentiated C2C12 myotubes were exposed to 10 μM dexamethasone (Dex) for 24 h in the presence or absence of CJE (10, 25, 50, and 100 μg/mL). Protein levels of FBX32 (Atrogin-1) and MuRF-1 were analyzed by Western blotting, with GAPDH as the loading control. **(A)** Representative Western blot images. **(B)** Densitometric analysis of protein expression normalized to GAPDH. Data are presented as mean ± SEM (n = 3). Statistical significance was determined using one-way ANOVA followed by Dunnett’s multiple comparisons test. *p < 0.05, **p < 0.01, ***p < 0.001 vs. control; #*p* < 0.05, ##*p* < 0.01, ###*p* < 0.001 vs. Dex.

To investigate the protective effects of CJE against glucocorticoid-induced muscle atrophy, differentiated C2C12 myotubes were treated with dexamethasone (10 μM) in the presence or absence of CJE. Representative Western blot images are shown in **Figure 5A**, while the corresponding densitometric analyses are presented in **Figure 5B**. Dexamethasone treatment increased the protein expression levels of FBX32 (Atrogin-1) and MuRF-1 compared with control cells. Co-treatment with CJE attenuated the dexamethasone-induced upregulation of these E3 ubiquitin ligases, with a more pronounced inhibitory effect observed for MuRF-1. Although the inhibitory pattern of FBX32 was not strictly concentration-dependent, overall FBX32 expression levels remained lower in the CJE co-treatment groups than in the dexamethasone-only group. These results suggest that CJE modulates key regulators associated with skeletal muscle protein degradation under glucocorticoid-induced atrophic conditions *in vitro* (Lee et al., 2022; Wu et al., 2022).

### 3.6 CJE attenuates dexamethasone-induced FOXO1 nuclear enrichment in C2C12 myotubes

**Figure 6:**
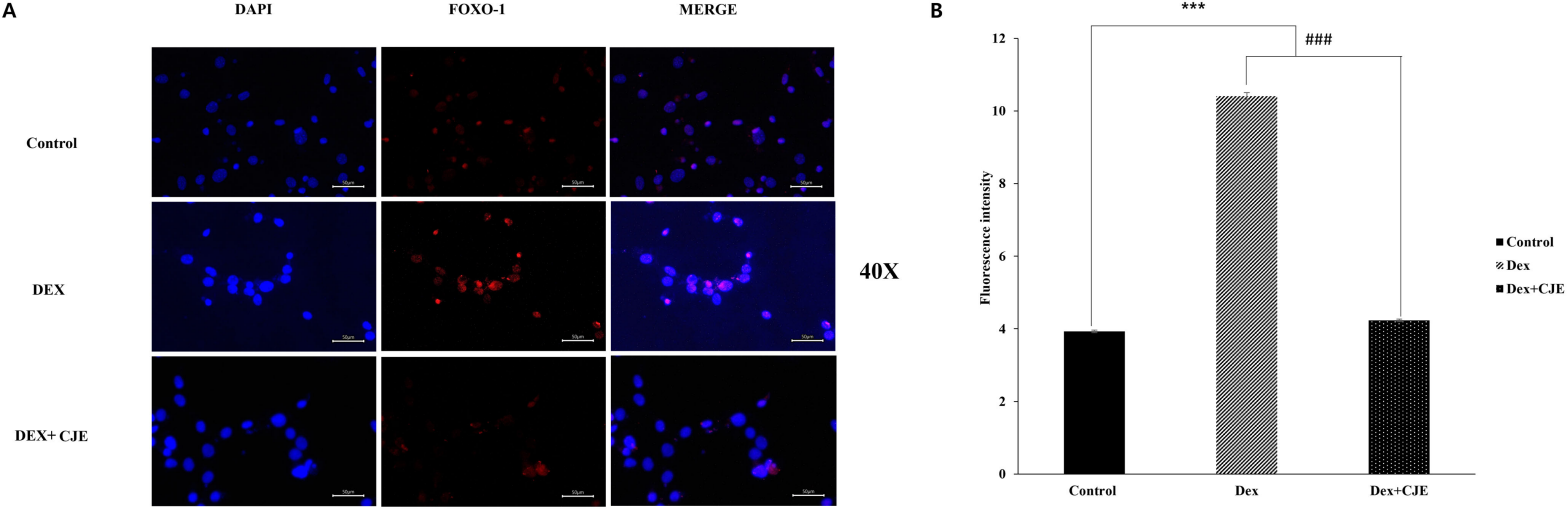
Qualitative visualization of FOXO1 distribution in dexamethasone-treated C2C12 myotubes and the modulatory effects of CJE. Differentiated C2C12 myotubes were treated with dexamethasone (Dex, 10 μM, 24 h) with or without CJE (100 μg/mL). Cells were fixed and immunostained with an anti-FOXO1 antibody (cat# 2880) followed by an Alexa Fluor 594 conjugated secondary antibody (cat# 8889; red) and counterstained with DAPI (blue). **(A)** Representative fluorescence images (40×) are shown. Dex treatment produced an apparent increase in FOXO1 signal within the nuclear region, whereas CJE co-treatment visibly reduced this Dex-associated nuclear enrichment. **(B)** Quantitative fluorescence analysis of FOXO1 signal within the nuclear area supports the qualitative trend observed in panel **(A)**. Data are expressed as mean ± SEM (n = 3). Scale bars = 50 μm. Statistical analyses were performed using one-way ANOVA followed by Dunnett’s *post hoc* test. ***p < 0.001 vs. control; ###p < 0.001 vs. Dex.

To FOXO1 acts as a key transcription factor that drives the expression of atrophy-related genes such as FBX32 when enriched within the nucleus (Lee et al., 2022; Wu et al., 2022). To explore whether CJE-mediated suppression of E3 ligases may involve modulation of FOXO1 distribution, we performed immunofluorescence imaging to visualize FOXO1 localization patterns in C2C12 myotubes. Dexamethasone treatment produced an apparent increase in FOXO1 signal within the nuclear region, as suggested by greater overlap with DAPI-stained nuclei (**Figure 6A**). In contrast, CJE co-treatment yielded a visibly reduced nuclear FOXO1 signal with a comparatively stronger cytoplasmic distribution. This qualitative observation was supported by the accompanying fluorescence intensity analysis, which showed decreased FOXO1 signal within the nuclear area following CJE co-treatment (**Figure 6B**). While immunocytochemical imaging provides supportive visualization rather than definitive mechanistic evidence, these findings collectively indicate that CJE diminishes dexamethasone-associated FOXO1 nuclear enrichment, thereby potentially reducing downstream activation of catabolic genes such as FBX32 (Wu et al., 2023b).

### 3.7 Effects of CJE on muscle fiber morphology and cross-sectional area

**Figure 7:**
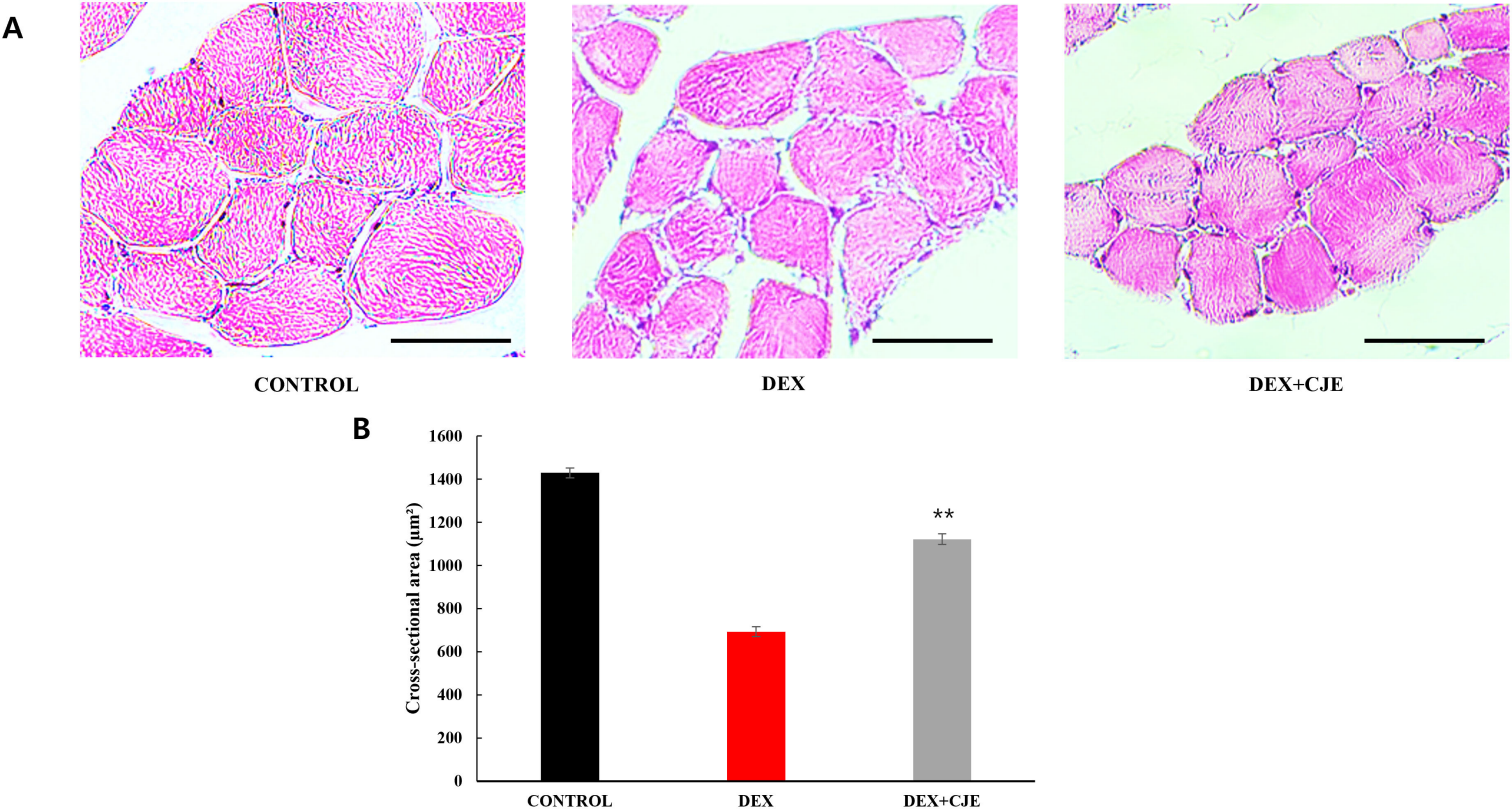
Effects of CJE on gastrocnemius muscle morphology and fiber size distribution in dexamethasone-treated mice. Representative hematoxylin and eosin (H&E) staining images of gastrocnemius muscle sections from **(A)** Control, DEX, and DEX + CJE groups (scale bar = 50 µm, 40×). **(B)** Quantification of mean CSA values (μm²). For each mouse, approximately 300 individual fibers were analyzed, and the mean CSA per mouse was treated as one biological replicate (n = 5 mice per group). Data are expressed as mean ± SEM. Statistical comparisons were performed using one-way ANOVA followed by Dunnett’s *post hoc* test. **p < 0.01 vs. Control.

To evaluate the structural effects of CJE on skeletal muscle, histological analysis of gastrocnemius muscle sections was performed using hematoxylin and eosin staining **Figure 7A**. Control mice exhibited normal muscle fiber architecture, characterized by polygonal fiber shapes and relatively uniform fiber size distribution **Figure 7B**. In contrast, dexamethasone treatment resulted in morphological alterations, including reduced muscle fiber cross-sectional area (CSA), increased variability in fiber size, and expanded interstitial spaces, consistent with muscle atrophy. Co-treatment with CJE attenuated these dexamethasone-induced histological changes, showing partial restoration of muscle fiber morphology and organization. Quantitative analysis further demonstrated that dexamethasone significantly reduced mean fiber CSA, whereas CJE treatment increased CSA compared to the dexamethasone group. These findings suggest that CJE mitigates glucocorticoid-induced muscle atrophy at the tissue level.

### 3.8 Effects of CJE on muscle atrophy–related signaling pathways *In Vivo*

**Figure 8:**
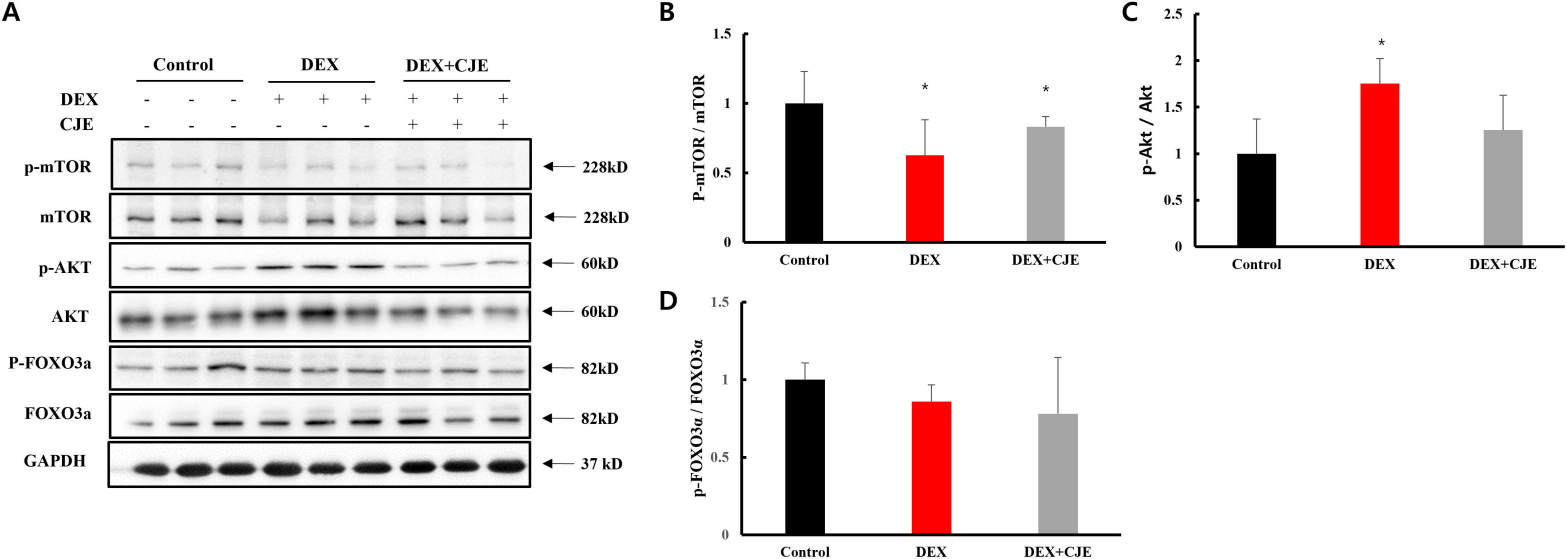
Effects of CJE on the expression of muscle atrophy-related signaling proteins in dexamethasone-treated mice. **(A)** Representative Western blot images from three mice per group showing the indicated proteins. GAPDH was used as a loading control **(B–D)** Densitometric analysis of mTOR, Akt, and FOXO3α protein expression normalized to GAPDH. Quantification was performed using all biological replicates (n = 4–6 per group). Data are presented as mean ± SEM. Statistical comparisons were performed using one-way ANOVA followed by Dunnett’s *post hoc* test. *p < 0.05 vs. Control.

Protein expression of key signaling molecules associated with muscle atrophy was analyzed in gastrocnemius muscle tissues (**Figure 8A**), and densitometric data are expressed as the ratio of phosphorylated to total protein (**Figures 8B–D**). Dexamethasone treatment significantly decreased the p-mTOR/mTOR ratio compared with control mice, and CJE co-treatment partially restored mTOR phosphorylation (**Figure 8B**). In contrast, the p-AKT/AKT ratio was significantly increased in the dexamethasone group relative to control and was reduced toward control levels following CJE co-treatment (**Figure 8C**). The p-FOXO3a/FOXO3a ratio did not differ significantly among the experimental groups (**Figure 8D**). Total AKT, mTOR, and FOXO3a protein levels remained relatively unchanged across all groups.

### 3.9 Effects of CJE on E3 ubiquitin ligase expression in skeletal muscle *In Vivo*

**Figure 9:**
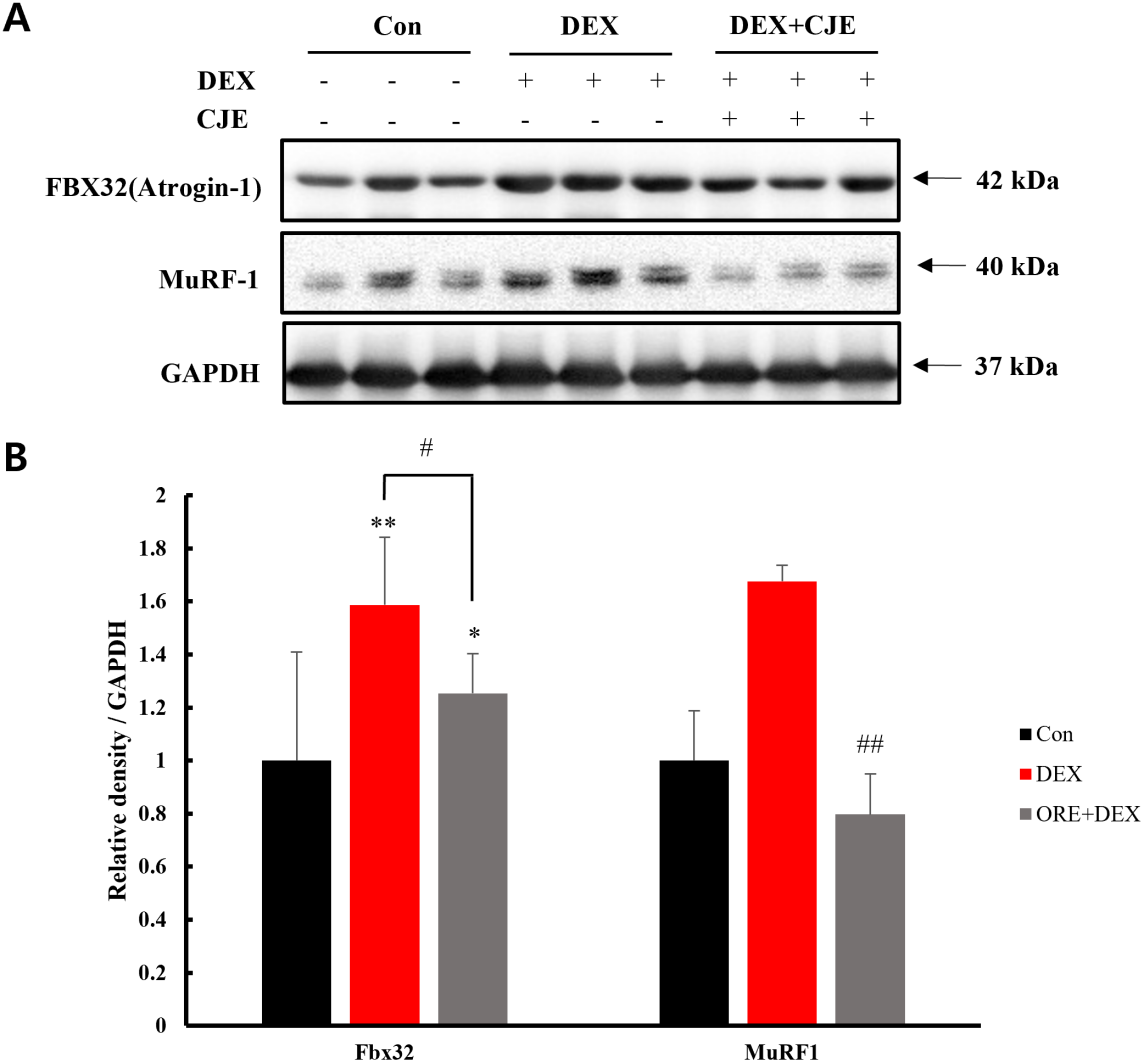
Effects of CJE on muscle atrophy-related E3 ubiquitin ligases in dexamethasone-treated mice. **(a)** Representative Western blot images from three mice per group showing FBX32 (Atrogin-1) and MuRF-1 expression. GAPDH was used as a loading control. **(b)** Densitometric analysis of FBX32 and MuRF-1 protein expression normalized to GAPDH. Quantification was performed using all biological replicates (n = 4–6 per group). Data are presented as mean ± SEM. Statistical significance was determined using one-way ANOVA followed by Dunnett’s multiple comparisons test. *p < 0.05, **p < 0.01 vs. Control; #*p* < 0.05, ##*p* < 0.01 vs. DEX.

Protein expression levels of FBX32 (Atrogin-1) and MuRF-1 in gastrocnemius muscle were analyzed by Western blot. As shown in **Figure 9A**, representative protein bands are presented, and **Figure 9B** shows the corresponding densitometric analysis. Dexamethasone administration increased the protein expression of FBX32 and MuRF-1 compared to control mice. Treatment with CJE reduced the expression levels of both proteins compared to the dexamethasone-treated group.

### 3.10 CJE modulates serum GDF-8 levels

As shown in **Table 1**, control mice exhibited baseline serum GDF-8 levels of 889.0 ± 47.9 pg/mL. Dexamethasone treatment significantly increased serum GDF-8 to 1304.0 ± 104.7 pg/mL. CJE co-treatment resulted in serum GDF-8 levels of 1051.5 ± 46.5 pg/mL, which was significantly lower than the Dex group, representing 80.6% of Dex levels. These results suggest that CJE modulates systemic factors involved in muscle protein metabolism and partially normalizes the dexamethasone-induced increase in this negative regulator of muscle mass.

**Table 1:** Effect of CJE on dexamethasone-induced changes in serum GDF-8 levels.

Group	GDF-8 (pg/mL; Mean ± SD)	% of Control	% of Dex
Control	889.0 ± 47.9 ##	100	68.2
Dex	1304.0 ± 104.7	146.7	100
Dex + CJE	1051.5 ± 46.5 #	118.3	80.6

GDF-8 concentrations were measured using an ELISA kit and expressed as mean ± SD (n = 5). #P < 0.05, ##P < 0.01 vs. Dex group.

## 4 Discussion

### 4.1 Principal findings

This study provides the first evidence that Cryptotaenia japonica extract (CJE) attenuates glucocorticoid-induced muscle atrophy through coordinated modulation of anabolic and catabolic signaling. Using complementary *in vitro* (C2C12 myotubes) and *in vivo* (ICR mice) models, CJE (1) attenuated Dex-induced upregulation of the E3 ubiquitin ligases Atrogin-1 (FBX32) and MuRF-1 in myotubes; (2) reduced Dex-associated FOXO1 nuclear enrichment; (3) partially restored Dex-suppressed mTOR phosphorylation *in vivo*; (4) downregulated Atrogin-1 and MuRF-1 in gastrocnemius muscle; (5) preserved muscle-fiber architecture and increased cross-sectional area (CSA); and (6) partially normalized the Dex-induced elevation of serum GDF-8. Together, these findings position CJE as a candidate natural agent against glucocorticoid-induced sarcopenia.

### 4.2 Modulation of anabolic signaling (mTOR/Akt)

CJE partially restored the Dex-suppressed p-mTOR/mTOR ratio *in vivo*, consistent with preservation of anabolic capacity, since mTORC1 is a central driver of muscle protein synthesis. The *in vivo* p-AKT response, however, diverged from the canonical IGF-1/Akt suppression model (Manning and Toker, 2017): the p-AKT/AKT ratio was elevated under Dex and was normalized toward control by CJE. This pattern may reflect a compensatory or feedback-mediated activation of Akt under sustained glucocorticoid exposure at the examined time point, which CJE attenuated. Accordingly, the anabolic recovery promoted by CJE was most consistently reflected by mTOR phosphorylation, whereas the *in vivo* Akt response should be interpreted cautiously given the single time point and the small group size (n = 3). These observations indicate that, in this model, mTOR rather than Akt phosphorylation is the more reliable anabolic readout of CJE activity.

### 4.3 Suppression of the FOXO/E3 ubiquitin ligase catabolic axis

The most consistent effect of CJE across *in vitro* and *in vivo* models was suppression of the FOXO/E3 ubiquitin ligase catabolic arm. In myotubes, CJE attenuated Dex-induced FOXO1 nuclear enrichment and the upregulation of Atrogin-1 and MuRF-1, and it reduced both ligases in gastrocnemius muscle *in vivo*. By contrast, the *in vivo* p-FOXO3a/FOXO3a ratio did not differ significantly among groups; the catabolic-suppressive effect of CJE *in vivo* was therefore most directly reflected by downregulation of the E3 ubiquitin ligases rather than by changes in FOXO3a phosphorylation. Because FOXO-driven Atrogin-1/MuRF-1 expression is a primary determinant of ubiquitin–proteasome–mediated myofibrillar breakdown (Zhao et al., 2007; Sandri, 2013), the consistent ligase suppression observed here provides a coherent mechanistic basis for the preserved fiber morphology and CSA.

### 4.4 Myostatin/GDF-8 regulation

CJE partially normalized the Dex-induced elevation of serum GDF-8 (myostatin), a TGF-β–superfamily negative regulator of muscle mass that promotes catabolic gene expression via Smad signaling and converges on the ubiquitin–proteasome system (McPherron et al., 1997; Zimmers et al., 2002; Lokireddy et al., 2012). The reduction of circulating GDF-8 by CJE suggests an additional, systemic contribution to muscle preservation that complements its direct suppression of the FOXO/E3 ligase axis.

### 4.5 Comparison with other natural extracts

The present findings align with reports that phytochemical-rich extracts counteract glucocorticoid-induced atrophy through the Akt/mTOR/FOXO network (Kim and Hwang, 2020; Nikawa et al., 2021; Wang et al., 2021; Lee et al., 2022). Asiatic acid alleviates Dex-induced atrophy via the Sirt1/PGC-1α/FOXO3 pathway (Ji et al., 2025), turmeric extract suppresses Dex-induced atrophy through modulation of protein-degradation pathways (Furukawa et al., 2022), and our previous work showed that *Quercus acuta* fruit extract acts through the IGF-1/Akt/FOXO axis (Choi et al., 2025). Notably, dieckol attenuates glucocorticoid-induced atrophy by suppressing NLRP3 inflammasome activation and pyroptosis (Oh et al., 2021). Given the emerging role of NLRP3 inflammasome-mediated inflammation in skeletal muscle atrophy and cachexia (Liu et al., 2022), inflammasome-driven inflammatory signaling may represent an additional therapeutic axis targeted by natural products. Whether CJE similarly modulates the NLRP3/caspase-1/GSDMD pathway warrants further investigation. Relative to these reports, CJE modulates a comparable breadth of catabolic readouts FOXO1 nuclear enrichment, E3 ubiquitin ligase expression, and circulating GDF-8 while its *in vivo* anabolic effect is most evident at the level of mTOR.

### 4.6 Phytochemical basis and potential role of chlorogenic acid

Among the constituents of CJE, chlorogenic acid was identified as a marker compound. Chlorogenic acid is a dietary polyphenol with well-characterized antioxidant and anti-inflammatory activities that are mechanistically relevant to muscle homeostasis. It has been reported to activate AMP-activated protein kinase (AMPK) and to modulate the PI3K/Akt/mTOR axis, which jointly govern the balance between protein synthesis and degradation in skeletal muscle (Wang et al., 2022). By attenuating oxidative stress and pro-inflammatory signaling both of which promote FOXO activation and E3 ubiquitin ligase induction chlorogenic acid may contribute to the suppression of catabolic gene programs observed with CJE (Gu et al., 2023). In addition, C. japonica contains caffeic acid, quercetin derivatives, and polyacetylenes (Lu et al., 2018), which may act additively or synergistically with chlorogenic acid. Because the present study did not isolate the active fraction, the contribution of chlorogenic acid remains associative; bioactivity-guided fractionation will be required to determine its relative role and to establish structure–activity relationships.

### 4.7 Limitations and future directions

Several limitations should be acknowledged. First, the active constituents of CJE were not isolated; bioactivity-guided fractionation and structural characterization are needed. Second, the upstream signaling by which CJE modulates Akt/mTOR and FOXO requires further elucidation, including IGF-1 receptor signaling, PI3K activation, and phosphatase activity (Rommel et al., 2001). Third, the *in vivo* analyses used a small group size (n = 3 for Western blotting) and a single muscle group (gastrocnemius) at one time point; larger cohorts, additional muscles (e.g., quadriceps and diaphragm), and time-course studies are warranted, particularly to clarify the divergent *in vivo* Akt response. Fourth, translational relevance requires validation in aged sarcopenia models and human studies.

## 5 Conclusion

This study provides evidence that *Cryptotaenia japonica* extract (CJE) exerts myoprotective effects against glucocorticoid-induced muscle atrophy. Using complementary *in vitro* and *in vivo* models, CJE restored mTOR phosphorylation, modulated AKT phosphorylation, attenuated FOXO1 nuclear enrichment, and reduced the expression of the E3 ubiquitin ligases Atrogin-1 (FBX32) and MuRF-1. In dexamethasone-treated mice, CJE further attenuated muscle fiber atrophy, preserved muscle architecture, and increased muscle fiber cross-sectional area. These findings suggest that CJE counteracts glucocorticoid-induced muscle wasting by coordinately regulating anabolic and catabolic signaling within the AKT/mTOR–FOXO/E3 ubiquitin ligase axis. Collectively, this study supports the potential of CJE as a natural therapeutic candidate for the prevention of sarcopenia and muscle-wasting conditions and warrants further investigation for clinical application.

## Data Availability

The data that support the findings of this study are available from the corresponding author upon reasonable request.
